# Impact of the COVID-19 Pandemic on Academic Productivity in Oncology: A Journal-, Conference- and Author-Level Analysis

**DOI:** 10.7759/cureus.65879

**Published:** 2024-07-31

**Authors:** Vivian S Tan, Andrew Warner, Anthony C Nichols, Eric Winquist, David A Palma

**Affiliations:** 1 Radiation Oncology, Western University, London, CAN; 2 Otolaryngology - Head and Neck Surgery, Western University, London, CAN; 3 Medical Oncology, Western University, London, CAN

**Keywords:** conferences, authorship, oncology, equity, covid-19 pandemic

## Abstract

This study assessed the impact of the coronavirus disease 2019 (COVID-19) pandemic on academic productivity in oncology, measured by conference abstracts, journal publications and individual authorship trends, using a reference time frame of 2018 to 2022. To assess overall academic productivity, data was obtained on the number of abstracts and articles submitted and published from a selection of oncology conferences and journals. To assess individual authorship patterns, 200 articles were randomly selected from 2018, and for the first or last authors, publications were tracked over subsequent years. Factors assessed included gender, continent, specialty, MD vs. non-MD and career status (early vs. late). The number of submitted and published conference abstracts trended downward over time between 2018 and 2022 (p=0.11 and p=0.16 respectively). Journal submissions increased to a peak in 2020 and then declined thereafter, but this did not translate into changes in the number of papers published. For the author-level analysis, factors significantly predictive of increasing publication rates in multivariable analysis were late career status (vs. early), clinician status (vs. non-clinician), surgery or public health/epidemiology specialty, and author located in Asia. Further research is needed to help ameliorate the impact of these disparities.

## Introduction

The coronavirus disease 2019 (COVID-19) pandemic presented substantial challenges to the delivery of medical care, especially in oncology [1-3]. Physicians reported that because of the pandemic, patients presented with more advanced disease, missed cancer screening and experienced more treatment interruptions [4]. In cancer research, there was a decrease in the initiation and enrollment of cancer clinical trials and challenges accessing wet lab/veterinary facilities for translational and basic science research [5-7]. 

Both within and outside of medicine, the influence of COVID-19 has not been equal across race, gender or socioeconomic status. For example, previous research has demonstrated that women were more likely than men to assume caretaking responsibilities during the pandemic, leading to widening of the gender gap [6]. The objective of this study was to determine the effect of the COVID-19 pandemic on academic productivity in oncology as measured by conference abstracts, journal publications and individual authorship trends. This article was previously presented as an oral presentation at the Canadian Association of Radiation Oncologists Annual Meeting in September 2023 and poster presentation at American Society for Radiation Oncology Annual Meeting in October 2023.

## Materials and methods

Conference abstracts

Using a reference time frame of 2018 to 2022, we sought to obtain the number of abstract submissions and abstract publications for a selection of conferences in oncology. Conferences included American Society of Radiation Oncology (ASTRO), European Society for Radiotherapy and Oncology (ESTRO), Canadian Association for Radiation Oncology (CARO), American Society of Clinical Oncology (ASCO), European Society for Medical Oncology (ESMO), ESMO Asia and Society of Surgical Oncology (SSO). The numbers of abstracts submitted each year were retrieved by contacting the conference organizers, and abstracts published were retrieved from conference websites/proceedings.

Journal submissions

We sought to obtain the number of submitted research articles and published research articles from 2018 to 2022 for a selection of 10 oncology journals. Journals included International Journal of Radiation Oncology*Biology*Physics (Red Journal), Radiotherapy and Oncology (Green Journal), Journal of Clinical Oncology (JCO), Lancet Oncology, Annals of Oncology, Journal of the National Cancer Institute (JCNI), Journal of the American Medical Association (JAMA) Oncology, JAMA Surgery, British Journal of Surgery (BJS) and Journal of the American College of Surgeons (JACS). The number of research articles submitted were retrieved by directly contacting journals, and articles published were retrieved from journal websites.

Conference/journals were selected from input from a radiation oncologist, a medical oncologist and a surgical oncologist to include perspectives from these specialties.

Authorship trends

To assess individual authorship patterns, we randomly selected 200 research articles from 2018 (i.e. the ‘index article’) from the journals above, and tracked publications over subsequent years for the first or last authors from that index article (for each index article first or last author was selected for a total of 100 first authors and 100 last authors to capture early and late career authors). Articles that were non-oncology related, or those that repeated a previously selected author, were replaced with another article randomly selected. For each selected author, the number of publications from 2018 to 2022 was abstracted from PubMed. Other factors retrieved from the article and/or an internet search included gender (based on scholarly profiles and Gender-API software (München, Germany) if needed [7-9]), MD vs. non-MD (used as a surrogate for clinician status), career status (early if within five years of training completion vs. late if beyond five years), specialty (i.e. radiation oncology, medical oncology, surgical oncology) and continent (i.e. Asia, Europe, North America, etc.).

Statistical analysis

Descriptive statistics were generated for conference abstracts, journal articles and authorship trends. Time trend analysis using univariable linear regression with year as a continuous predictor (linear trend test) was performed to assess changes in number of conference abstracts and journal articles over time. In addition, linear time trend analyses and independent two-sample t-tests were used to assess changes in author productivity over time, while univariable and multivariable linear regression were used to identify individual factors predictive of publication rates. All eligible variables were incorporated into a multivariable regression model and sequentially removed using backward elimination techniques until all remaining covariates had p-values < 0.05. All statistical analysis was performed using SAS version 9.4 software (SAS Institute, Cary, NC, USA). 

## Results

Conference abstracts

**Figure 1:**
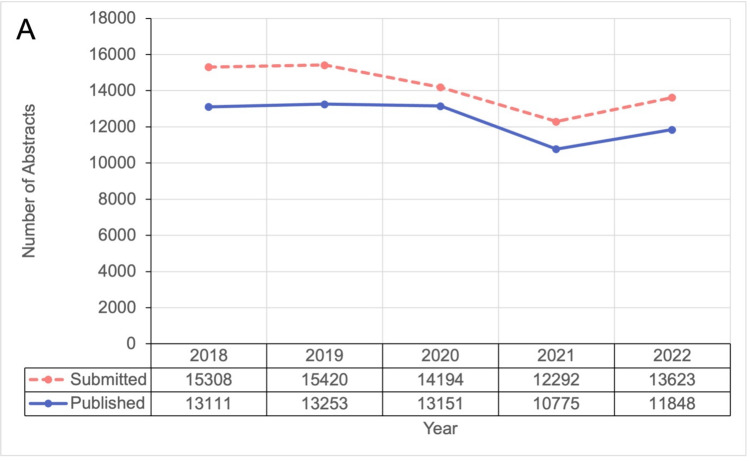
Number of abstract submissions and publications by year from 2018 to 2022. Note submissions were collected from five conferences, publications were collected from seven conferences.

Of the seven conferences selected a priori, data on submitted abstracts were available from five. Conference submissions trended downward over time, with 15,308 in 2018, 15,420 in 2019, 14,194 in 2020, 12,292 in 2021 and 13,623 in 2022 (p=0.11; Figure 1). Published conference abstracts from the seven selected conferences followed a very similar trajectory trending downward over time, from 13,111 in 2018 to 11,848 in 2022 (p=0.16; Figure 1). All seven conferences were converted to online in 2020. In 2021, five conferences remained online, one conference returned to in-person and one conference was cancelled. All seven conferences returned to in-person in 2022.

Journal submissions

**Figure 2:**
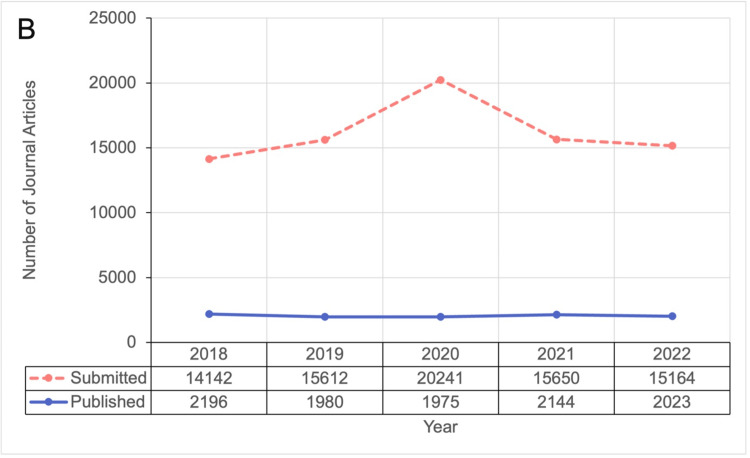
Number of journal article submissions and publications by year from 2018 to 2022. Note submissions were collected from six journals, publications were collected from 10 journals.

Journal submissions were available from six journals and increased from 14,142 in 2018 to a peak of 20,241 in 2020 (2018 vs. 2020: p<0.001), and then declined to 15,164 in 2022 (Figure 2). The total number of published articles from 10 journals showed no clear trends over time, with a slight decrease after 2020 (p=0.64; Figure 2).

Authorship trends

For the author-level analysis, of the 200 randomly selected authors, the majority were male (66.5%, n=133), from North America (55.5%, n=111), with an MD degree (80.9%, 131/162) and late career (86.6%, 129/149). The most common specialties included surgery (29%, n=58), radiation oncology (18.5%, n=37), epidemiology/public health (11%, n=22) and medical oncology (10%, n=20). In terms of articles authored per year, there was no significant linear trend detected across the whole group (p=0.51), although the median (interquartile range) number of publications per author peaked in 2020/2021 (articles/year for 2018-2022: 10 (5-20), 10 (4.5-21), 10.5 (5-22.5), 11 (4-24), nine (3-22)).

On univariable analysis, factors significantly associated with increasing publication rates were male gender, last author position on index article, late career status, MDs, speciality of surgery or public health/epidemiology, and authors located in Asia (all p<0.01) (Table 1). On multivariable analysis, factors remaining significantly predictive were late career status, MDs, surgeons, public health/epidemiologists, and authors located in Asia (all p<0.01) (Table 1).

**Table 1:** Univariable and multivariable linear regression models predictive of number of articles in author-level analysis Abbreviations: RC – regression coefficient; SE – standard error; P-values < 0.05 shown in bold ^1^All univariable models adjusted for year as predictor (RC ± SE not shown for year) ^2^Not considered for multivariable modelling

Variable:	Univariable^1^		Multivariable	
	RC ± SE	p-value	RC ± SE	p-value
Year (per 1 year), n=200	0.60 ± 0.92	0.51	0.60 ± 0.88	0.49
^2^Last vs. first author, n=200	8.53 ± 2.58	0.01	--	--
Male vs. female, n=199	9.67 ± 2.75	< 0.01	--	--
Specialty (vs. other), n=200	--	< 0.01	--	< 0.01
Epidemiology / public health	12.70 ± 5.10	0.10	20.64 ± 5.21	< 0.01
Hematology	10.31 ± 6.21	0.05	3.08 ± 6.15	0.62
Medical Oncology	10.11 ± 5.25	0.18	6.19 ± 5.14	0.23
Oncology	7.01 ± 5.17	0.37	0.04 ± 5.13	> 0.99
Radiation Oncology	3.98 ± 4.47	< 0.01	3.05 ± 4.35	0.48
Surgery	16.84 ± 4.09	< 0.01	15.31 ± 4.08	< 0.01
Continent (vs. Asia), n=200	--	< 0.01	--	< 0.01
Europe	-16.84 ± 4.10	< 0.01	-16.79 ± 4.16	< 0.01
North America	-17.44 ± 3.78	< 0.01	-25.08 ± 4.15	< 0.01
Clinical vs. non-clinical education, n=162	12.19 ± 3.98	< 0.01	16.52 ± 4.06	< 0.01
MD degree (vs. no), n=162	12.19 ± 3.98	< 0.01	--	--
MSc degree (vs. no), n=162	2.53 ± 3.75	0.50	--	--
PhD degree (vs. no), n=162	-0.73 ± 3.22	0.82	--	--
^2^Degree (vs. MSc), n=162	--	< 0.01	--	--
MD	23.83 ± 13.88	0.09	--	--
MD + MSc	12.57 ± 14.30	0.38	--	--
MD + MSc + PhD	79.18 ± 16.20	< 0.01	--	--
MD + PhD	18.63 ± 14.14	0.19	--	--
MSc + PhD	19.90 ± 15.31	0.19	--	--
PhD	8.17 ± 14.33	0.57	--	--
Late vs. early career status, n=149	14.46 ± 2.42	< 0.01	12.43 ± 4.36	< 0.01
Gender + career status (vs. female + late), n=149	--	< 0.01	--	--
Female + early	-12.82 ± 4.74	< 0.01	--	--
Male + early	-9.86 ± 3.01	< 0.01	--	--
Male + late	5.72 ± 1.88	< 0.01	--	--

## Discussion

To our knowledge, this is the first assessment of the impact of the pandemic on journal and conference submissions and publications in oncology. Our study demonstrated an increase in journal submissions in 2020, which is also reflected in previous studies in other specialties. An analysis of the neurosurgical, stroke neurology and neurointerventional literature found a 42.3% increase in original submissions in 2020 [10]. In addition, an analysis from 11 participating journals from the BMJ found that manuscript submissions in 2020 increased by 18.6% when compared to 2019 and by 23.3% when compared with 2018 [8]. Author in a surgical specialty was also a significant predictor of increasing publication rates in our univariable and multi-variable analyses. These findings may reflect that cancellations in clinical schedules and surgical procedures allowed more time for academic output for some researchers.

We found disparities in publication trends. These included findings in univariable analysis that academic productivity decreased to a greater degree among women than in men during the pandemic. Previous studies have shown that women submitted fewer articles as compared to men early in the pandemic [6,8,9,11,12]. In a study published in the BMJ examining female authorship of COVID-19 research, women were less represented in first, last and corresponding authorship than compared to prior to the pandemic [8]. There are a number of factors that may play a role including discrimination against women in the workplace, institutional barriers, higher burden of invisible work (i.e. childcare responsibilities), underrepresentation of women in leadership positions and others. However, we recognize our study is only able to comment on publication trends and further exploration of factors associated with the gender disparity is out of the scope of the current study. We support further work to continue to explore inequality in academia. In addition, in multivariable analysis early-career researchers demonstrated decreased academic productivity as compared to more senior researchers. Increased clinical demands may have decreased research exposure and closures of universities and research institutions leading to transition to work from home leading to less in-person mentorship for trainees and early-career researchers [5]. Our study also demonstrated a decreasing trend in conference abstracts, however this could have been confounded by decreased travel that was heavily influenced by the pandemic.

This study has several limitations, including that only a sample of oncology conferences and journals were used to represent oncology productivity as a whole. Conference and journal publications were retrieved from publicly available data which was complete. However, submissions data relied on contacting conferences and journals directly. Not all contacted conferences and journals provided their submission data. Consequently, there may be risk of bias. In addition, details regarding submission characteristics such as article type and author features were not able to be retrieved. Authorship trend analysis relied on random sampling as it was impractical to collect authorship data for all authors who published during the time period. Factors such as career status were only available in a subset of authors from public data. In our analysis of the impact of gender, there was no ability to identify transgender or non-binary authors, and although we estimated gender using standardized approaches adopted from other studies, some misclassification could occur.

## Conclusions

The COVID-19 pandemic was associated with downward trends in conference submissions and acceptances from 2018 to 2022. Journal submissions peaked overall in 2020, but this did not translate to increases in the overall number of papers published. Disparities in publication trends were found, based on speciality and geographic regions, including a negative impact on early-career researchers. Further research is needed to ameliorate disparities from those disproportionately affected.
